# (Quasi)-Binomial vs. Gaussian Models to Evaluate Thiamethoxam, Pirimiphos-Methyl, Alpha-Cypermethrin and Deltamethrin on Different Types of Storage Bag Materials Against *Ephestia kuehniella* Zeller (Lepidoptera: Pyralidae) and *Tribolium confusum* Jacquelin du Val (Coleoptera: Tenebrionidae)

**DOI:** 10.3390/insects12020182

**Published:** 2021-02-21

**Authors:** Nikos E. Papanikolaou, Nickolas G. Kavallieratos, Maria C. Boukouvala, Chrisovalantis Malesios

**Affiliations:** 1 Laboratory of Agricultural Zoology and Entomology, Department of Crop Science, Agricultural University of Athens, 75 Iera Odos str., 11855 Athens, Greece; nick_kaval@aua.gr (N.G.K.); mbouk@aua.gr (M.C.B.); 2 Directorate of Plant Produce Protection, Greek Ministry of Rural Development and Food, 150 Sygrou Ave., 17671 Athens, Greece; 3 Laboratory of Political Economy and European Integration, Department of Agricultural Economics and Rural Development, Agricultural University of Athens, 75 Iera Odos str., 11855 Athens, Greece; malesios@aua.gr

**Keywords:** thiamethoxam, pirimiphos-methyl, alpha-cypermethrin, deltamethrin, Mediterranean flour moth, confused flour beetle, storage bag materials, mortality rate, modeling

## Abstract

**Simple Summary:**

*Ephestia kuehniella* Zeller (Lepidoptera: Pyralidae) and *Tribolium confusum* Jacquelin du Val (Coleoptera: Tenebrionidae) are major stored-product pests globally. The use of contact insecticides constitutes an option of effective management of these species. Therefore, we evaluated the activity of thiamethoxam, pirimiphos-methyl, alpha-cypermethrin, and deltamethrin for the management of *E. kuehniella* and *T. confusum* larvae on three types (plastic and paper) of storage bag materials under three exposure scenarios. In addition to typical linear regression, we introduced a binomial and quasi-binomial modeling approach to analyze our data. Thiamethoxam and pirimiphos-methyl exhibited significantly higher mortality rates on *E. kuehniella* and *T. confusum* than alpha-cypermethrin and deltamethrin. Mortality rate of *T. confusum* larvae was significantly higher than *E. kuehniella* larvae. Treatments revealed significant differences in the mortalities of both species. Our results indicate that the application of contact insecticides to storage bag materials is an efficient management tool against larvae of *E. kuehniella* and *T. confusum*.

**Abstract:**

The Mediterranean flour moth, *Ephestia kuehniella* Zeller (Lepidoptera: Pyralidae) and the confused flour beetle, *Tribolium confusum* Jacquelin du Val (Coleoptera: Tenebrionidae) are worldwide spread and notorious organisms of numerous stored-products. Both species are dangerous for bagged commodities as penetrators and invaders. The aim of the current study was to examine the efficacy of thiamethoxam, pirimiphos-methyl, alpha-cypermethrin, and deltamethrin, against *E. kuehniella* and *T. confusum* larvae, on different types of storage bag materials, i.e., woven propylene, biaxially oriented polypropylene and kraft paper through a (quasi)-binomial modeling approach. The type of the tested storage bag material did not affect the mortality rates of both species when treated with the tested insecticides. Thiamethoxam and pirimiphos-methyl showed statistically significant higher mortality rates on *E. kuehniella* and *T. confusum* (beta coefficient = 0.141; *p*-value < 0.05) compared to alpha-cypermethrin and deltamethrin. In addition, *T. confusum* exhibited significantly higher mortality rate in comparison to *E. kuehniella*. Our results also showed that the tested doses and surface treatments had a significant effect on the mortality *E. kuehniella* and *T. confusum* larvae. Significantly higher mortality rates were recorded when larvae were exposed on bag materials having both surfaces treated or on the single treated surface than when they were exposed on the untreated surface. Our findings can be useful towards an effective management strategy against stored-product insect pests.

## 1. Introduction

Insect pests threaten the commercial and nutritional value of several agricultural stored-products [1,2,3,4,5,6,7,8]. In particular, the quality and quantity of stored-products in storage facilities are considerably downgraded due to insect infestations [9,10]. According to the Food and Agriculture Organization (FAO) of the United Nations, the qualitative and quantitative degradation of stored-products by insect pests amounts to 10% of the world production in developed countries and exceeds 20% in developing countries [11]. The majority of the stored-product insect pests, as well as the most notorious species, belong to the orders of Lepidoptera and Coleoptera [12,13,14].

The efficient storage of agricultural products can reduce insect pest activity, as well as their dispersion through transportation, contributing to the preservation of their qualitative and quantitative characteristics [15]. Packaging of stored products in storage bags (e.g., polypropylene, polyethylene, polyester, multilayer) is a common method that prevents insect infestation [2,16,17,18,19,20]. However, this practice is not always sufficient. Human activities (e.g., mechanical handling, not proper sealing) as well as the ability of insect pests to penetrate the packaging material or invade the bagged stored products through existing openings often make this method ineffective [15,21]. Treating storage bags with insecticides or insect growth regulators provides an alternative method towards an efficient management of stored-product insect pests [19,22,23,24,25]. This method offers certain advantages since it reduces the infestation of bagged stored products by insect-pests and their introduction into a new environment due to transportation. Furthermore, it reduces the escape of insects which developed inside bags, thus preventing establishment of new infestations to adjacent bulked or bagged commodities [15,18,19,22,25,26]. 

The presence of the Mediterranean flour moth, *Ephestia kuehniella* Zeller (Lepidoptera: Pyralidae), in storage facilities is common worldwide [27,28,29,30,31]. Larvae of this species infest amylaceous commodities, such as cereals and various types of flours, nuts and legumes, dried vegetables, sugar, leaves of tobacco, dead insects, and milk [1,32,33,34,35,36,37]. Apart from direct damages, larvae cause indirect quantitative degradations on the infested stored products due to their feces and webbings [37,38]. The confused flour beetle, *Tribolium confusum* Jacquelin du Val (Coleoptera: Tenebrionidae), is a cosmopolitan pest of agricultural stored products [11,39]. It infests 138 different commodities such as cereal seeds, flours, bran, oilseeds, spices, as well as a wide variety of dry plant materials (e.g., fruits, nuts, herbs, coffee) [34]. It is considered as one of the most important enemies of stored cereals and processed cereals in a wide spectrum of storage facilities (e.g., mills, bins, bakeries) [1,34,40,41,42]. The qualitative and quantitative degradation of stored products in places where *T. confusum* occurs is particularly important, taking into account the significant financial losses on the basis of an annual global scale [11,39]. 

Pyralidae and Tenebrionidae are categorized as penetrators and invaders, i.e., they infest bagged commodities by making holes in packaging materials and by entering through formed apertures of the packages, respectively [20,21]. This issue becomes more serious when species of both families, such as *E. kuehniella* and *T. confusum*, coexist in facilities where the commodities are stored in bags. Considering also the elevated economic importance of *E. kuehniella* and *T. confusum* as stored-product insect pests, a study was initiated in order to examine the efficacy of four common insecticides, i.e., thiamethoxam, pirimiphos-methyl, alpha-cypermethrin, and deltamethrin, against these notorious species on different types of storage bag materials. For this purpose, woven and biaxially oriented polypropylene bags, as well kraft paper bags were used as packaging materials. To assess the impact of the explanatory variables (i.e., insecticides, insect species, doses, and surface treatments) on insect mortality rate, a binomial and quasi-binomial modeling approach was utilized, in addition to the typical Gaussian regression model, given that it serves as an efficient tool to analyze this type of data [43]. For this purpose, the sample mortality rate, as collected from this study, is utilized and treated as response data in the aforementioned regression models and regressed upon the various explanatory variables. The derived equation that best describes the variability in the response variable of mortality rate can be subsequently used for the estimation of mortality rate in the population of the two species, based upon the specific explanatory variables.

## 2. Materials and Methods

### 2.1. Insects

Colonies of *E. kuehniella* and *T. confusum* were initiated in 2003 at the Laboratory of Agricultural Entomology, Benaki Phytopathological Institute from individuals that had been collected from Greek storage facilities. Since 2014, the colonies were kept at the Laboratory of Agricultural Zoology and Entomology, Agricultural University of Athens. *Ephestia kuehniella* was reared on wheat flour at 25 °C and 65% relative humidity in continuous darkness. *Tribolium confusum* was reared on wheat flour with the addition of 5% brewers’ yeast (w/w) at 25 °C and 65% relative humidity in continuous darkness. *Ephestia kuehniella* and *T. confusum* 3rd–4th instar larvae [44] were selected for the experiments.

### 2.2. Insecticides

Four insecticidal formulations were used in the tests: Actara WG that contains 250 g/kg thiamethoxam active ingredient (a.i.), Actellic EC that contains 50% pirimiphos-methyl (a.i.) (provided by Syngenta, Anthousa, Greece), Power SC that contains 62.4 g/L alpha-cypermethrin (a.i.) (provided by Hybrid Hellas, Metamorphossis, Greece), and K-Othrine WG that contains 25% deltamethrin (a.i.) (provided by Bayer Hellas, Amaroussion Greece).

### 2.3. Storage Bag Materials

Three types of bag materials were used in the tests: woven polypropylene bags (Hatzigeorgiou-Fakaros G.P., Aigaleo, Greece), that are recommended for the storage of grains, olives, cotton, charcoal, and wood (a); biaxially oriented polypropylene bags (Alpha Beta Roto S.A., Aigaleo, Greece), that are recommended for the storage of grains, human or animal foods (b); kraft paper bags (Mondi, Vienna, Austria), that are recommended for the storage of grains, fresh fruits and vegetables, fish or meat (c). The technical characteristics of these bags are shown in Table 1. 

### 2.4. Experimental Set-Up

The insecticides were tested as surface treatments at the following doses: 0.025, 0.05, and 0.10 mg (a.i.)/cm^2^ in Petri dishes (8 cm diameter by 1.5 cm height). In order to facilitate the robustness and consistency of inference and corresponding estimations of statistical regression modeling, the doses were kept under the same scale conditions for all the examined insecticides. The surface area of each dish was 50.27 cm^2^. All types of bags were cut in circular pieces to cover the internal bottoms of the dishes, as it has been suggested in numerous recent studies concerning packaging materials. For example, Arthur [23] evaluated the impact of different storage bag materials treated with methoprene by exposing larvae and adults of the red flour beetle, *Tribolium castaneum* and *T. confusum* to circular pieces of these materials attached on the bottom of Petri dishes. Later, Scheff and Arthur [24] estimated the impact of deltamethrin-impregnated bag material to the fecundities of *T. castaneum* and *T. confusum*. For this purpose, adults of both species were exposed to circular sections of the bag material attached on the bottom of Petri dishes. Scheff et al. [25] determined the effect of packaging material treated with methoprene with the exposure of eggs or larvae of the Indianmeal moth, *Plodia interpunctella* (Hübner) (Lepidoptera: Pyralidae) and the warehouse beetle, *Togoderma variabile* Ballion (Coleoptera: Dermestidae) to semicircular pieces of the material stuck on Petri dishes. Furthermore, Scheff et al. [26] measured the impact of packaging material that had been treated with methoprene to larvae of *T. castaneum* and *T. variabile* under different exposure scenarios on pieces of this material cut in circles attached on the bottom of Petri dishes. The bag materials were sprayed with a fine mist of 1 mL of an aqueous solution which contained the appropriate volume of thiamethoxam, pirimiphos-methyl, alpha-cypermethrin, and deltamethrin corresponding to each tested dose. An AG-4 airbrush (Mecafer S.A., Valence, France) was used to spray the bag materials as follows: (a) one surface was sprayed, (b) both surfaces were sprayed. After each insecticidal application the airbrush was carefully cleaned with acetone and then next treatment was conducted. An additional series of bag materials were prepared and sprayed with distilled water as described above and served as controls. A different AG-4 airbrush that is reserved for controls was used. The treated bag materials were left to dry for 24 h at 25 °C and 65% relative humidity. Then, the bag materials with both surfaces treated were affixed to the bottom of the dishes whereas the bag materials with one surface (outside) treated were affixed to the bottom of the dishes, having the treated surface either facing up or facing down. Each bag material was adhered to the dish by using a white sealant (Zwaluw Star Acryl, Den Braven, Oosterhout, The Netherlands) that was placed only along the border of the bag material and the dish. The upper internal sides of the dishes were coated by polytetrafluoroethylene (60 wt % dispersion in water) (Sigma-Aldrich Chemie GmbH, Taufkirchen, Germany) to prevent insects from escaping. Ten larvae of *E. kuehniella* or *T. confusum* were transferred to each dish. The covers of the dishes had each a 1.5 cm circular aperture covered by muslin (perforated material) to allow the adequate ventilation of the internal part of the dishes. The pieces of muslin cloths were attached on the covers of the dishes by using a transparent sealant (SEL-AQ-300, Selsil, Esenler, Turkey). Dishes were placed inside incubators set at 25 °C, 65% relative humidity and continuous darkness. Mortality was evaluated by prodding larvae gently with a brush to detect any movement under an Olympus stereomicroscope (Olympus SZX9, Bacacos S.A., Athens, Greece) 1 h, 2 h, 3 h, 1 day, 2 days, 3 days, and 10 days post-exposure. Different brushes (Cotman 111 No 000, Winsor and Newton, London, UK) were used for the examination of insect individuals per species and treatment. The tests were conducted in a completely randomized block design with three subreplicates and three replicates.

### 2.5. Statistical Methodology

#### 2.5.1. Regression-Type Modeling

In order to effectively examine the combined effects of factors such as insect species (two categories: (1) *T. confusum*, (2) *E. kuehniella*); insecticide (five categories: (1) thiamethoxam, (2) pirimiphos-methyl, (3) alpha-cypermethrin, (4) deltamethrin, (5) control); type of surface bag material (three categories: (1) woven polypropylene, (2) biaxially oriented polypropylene, (3) kraft paper); treatment (three categories: (1) one surface was treated, insects were exposed to treated surface, (2) one surface was treated, insects were exposed to untreated surface, (3) both surfaces were treated) and dose (three categories: (1) 0.025 mg (a.i.)/cm^2^, (2) 0.05 mg (a.i.)/cm^2^, (3) 0.10 mg (a.i.)/cm^2^), on the percent mortality rate of larvae (dependent variable), a series of alternative regression type models are fitted to the collected sample of data (*n* = 16,740). The previously described covariates constitute the fixed-effects terms of the fitted regression models. In addition, the fitted models include as a random effect term the exposure time of the larvae on the insecticides in terms of mortality rate.

In particular, the following general formula for the regression modeling was fitted to the collected data:

*g(μ_i_) = β*_0_*+ β*_1_*· (pirimiphos-methyl) + β*_2_*· (alpha-cypermethrin) + β*_3_*· (deltamethrin) + β*_4_*· (control) + β*_5_*· (biaxially oriented polypropylene) + β*_6_*· (kraft paper) + β*_7_*· (E. kuehniella) + β*_8_*· (dose = 0.05 mg (a.i.)/cm^2^) + β*_9_*· (dose = 0.10 mg (a.i.)/cm^2^) + β*_10_*· (treatment = one surface was treated, insects were exposed to untreated surface) + β_11_ · (treatment = both surfaces were treated) + ε_i_ + η_i_*

where *μ_i_ = E(y_i_*) (*i* = 1, 2, …, 16,740) denotes the average mortality rate, *β*_0_ is the intercept, and *β_j_* (*j* = 1, 2, …, 11) are the regression coefficients for the various levels of the categorical covariates in the regression equation. Further, g(⋅) in the previous equation is the link function, linking the average mortality rate with the covariates, suitably specified for the various distributional specifications. With *ε*_i_ and *η*_i_ is denoted the error term and the random effect term of exposure, respectively. For the categorical covariates, the first level of each one has been assigned as the reference category for statistical comparisons between the various levels. 

**Figure 1:**
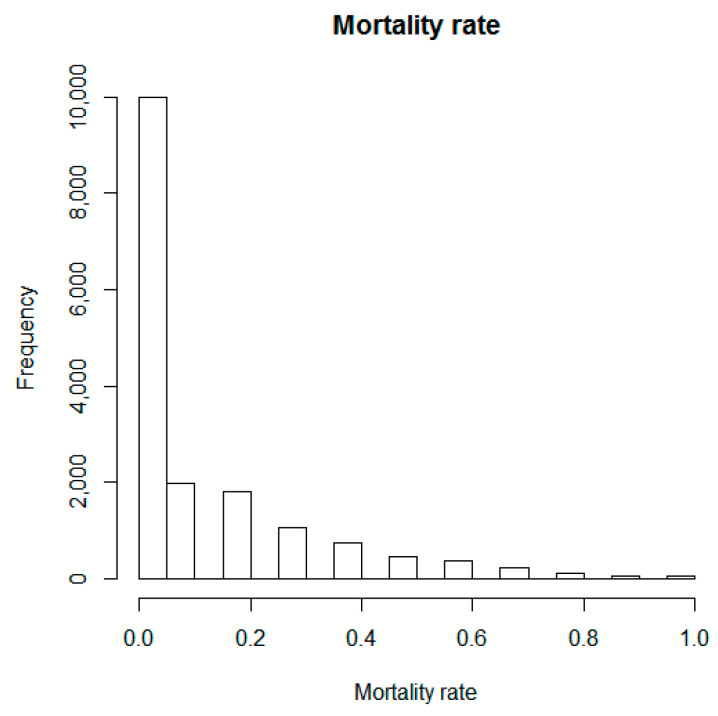
Frequency plot of the response variable of mortality rate (%).

Regarding the selection of a suitable link function for the above equation, and due to the nature of the response variable, being a proportion restricted to the interval between 0 and 1 (see Figure 1), besides the typical approach of assuming a Gaussian distribution linking the response and the covariates, we additionally fitted the data utilizing a binomial regression model (specifically we utilized the logit link function).

Further to the previous two modeling specifications (Gaussian and binomial), a quasi-binomial regression model [45,46] was applied to the data, linking the response variable to the selected covariates. The quasi-binomial model theoretically can better correspond to empirical response data for which their variance is larger (over dispersion) or smaller (under dispersion) than one gets with binomial models. The logit link function was also utilized for the quasi-binomial specification.

The binomial and quasi-binomial models were selected due to the special nature of the dependent variable that are proportions restricted to the interval between 0 and 1. Treating a proportional response as a continuous Gaussian variable may lead to severe problems and distortion of results in the case of regression modeling [47]. Another disadvantage of the Gaussian linear regression models is that they correspond to predictions below 0 or above 1, which is inappropriate for this type of data.

#### 2.5.2. Model Comparison and Covariate Selection

Model selection in terms of the different distributional specifications for describing the collected data (Gaussian, binomial, quasi-binomial) was performed by visual inspection of plots of predicted vs. observed responses, as well as by utilizing the mean square prediction error (*MSPE*) for each model. Empirical *MSPE* is calculated by the following formula:

(1) $$MSPE=E\left[{\left(y_{i}-{\hat{y}}_{i}\right)}^{2}\right],$$

where $y_{i}$ corresponds to the empirical values of the response variable of mortality rate and ${\hat{y}}_{i}$ are the predictions obtained by the fitted regression models. Lower values for the *MSPE* indicate the best fit to the data.

Subsequently, upon electing the best distributional specification, we applied covariate selection in order to avoid multicollinearity issues. Covariate selection is based upon a nested model comparison approach, which consists of the comparison between the null model (denoted by Model 1) including as fixed-effects predictors only the grand mean, i.e.,

(2) $$g\left(\mu_{i}\right)=\beta_{0}+\varepsilon_{i}\;(\mathrm{null}\;\mathrm{Model}\;1)$$

and the full model (adding one new predictor variable at each step). The overall significance of each model is then evaluated through the deviance statistic (based on the likelihood of each model) and model comparison is based on the likelihood ratio test (*LRT*) comparing each time the initially fitted model (null model) with the alternative model including an additional parameter [48]. The *LRT* statistic is calculated as:

(3) $$D=2\times \left(\ln(likelihood_{Mi})-\ln(likelihood_{Mi-1})\right)$$

where with *M_i_*_−1_ we denote the reduced model and with *M_i_* the model with the additional independent variable. The best model is the one showing the lowest levels of *D*.

Especially for the case of the quasi-binomial regression models, selection is based upon the quasi-likelihood counterpart of Akaike information criterion (*AIC*), namely *QAIC* statistic [49]. The best model in terms of model fit is the one with the lowest *QAIC* value.

#### 2.5.3. Model Fit

The R software [50] was utilized for fitting the regression models. The lme4 library [51] was used to fit the linear (Gaussian) models. In particular, for the fitting of the linear models we used the lmer() function, whereas the glmer() function was used for the fit of the binomial-type models (i.e., the binomial and the quasi-binomial regression models).

## 3. Results

**Figure 2:**
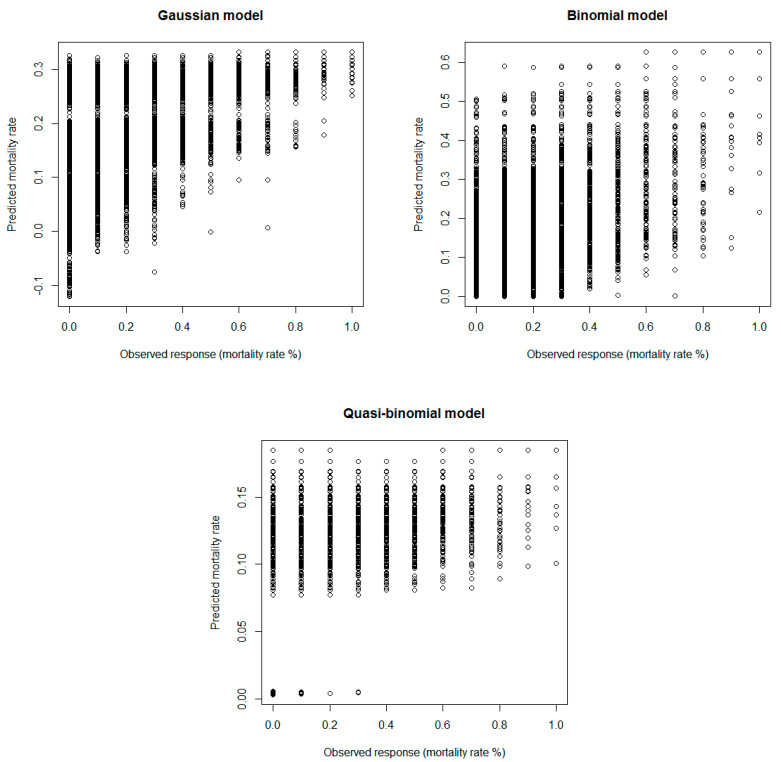
Observed response (mortality rate %) vs. predicted mortality rate for the Gaussian, binomial and quasi-binomial best fitted models.

The results for model comparison and covariate selection for the mortality rate data are summarized in Table 2 and Figure 2. Specifically, Table 2 includes the results of *LRT* for all three distributional specifications, utilized for the covariate selection. Most of the independent variables are important for the explanation of the variations in the mortality rates. In the case of the Gaussian regression models, we observed that all covariates are important, whereas in the two binomial-type models the covariate of surface type is the only one not statistically significant. The mean square prediction error is presented, indicating that the best distributional specification is the one of the quasi-binomial regression model (*MSPE* = 0.0132). This result is also verified by the inspection of predicted vs. observed response data (Figure 2). Inspection of the three graphs clearly shows the closest fit between the observed and predicted mortality rates of the quasi-binomial regression model. On the other hand, the worst fit seems to be the one of the binomial model (*MSPE* = 0.0217). This finding may be directly related to the presence of over dispersion in the response variable of mortality rate, since the empirical variance of mortality rate ($s^{2}=0.03$) is higher than the estimated variance from the binomial model (${\hat{s}}_{binomial}^{2}=0.01$).

Hence, upon selecting the quasi-binomial as the best distributional specification, next we utilized the deviance and corresponding *LRT* to select the most statistically significant covariates. The values of the deviance and corresponding *p*-values of the *LRT* for the quasi-binomial alternative nested models (Table 2) point out the best fitted model, including the random effects of exposure time, and fixed-effects covariates of insecticide, insect species, dose and treatment (*D* = 4698.3; *p*-value < 0.01).

The parameter estimate results of the best fitted quasi-binomial regression model, along with the corresponding 95% confidence intervals for the latter are summarized in Table 3.

The insecticide was an important factor that determined the mortality rate levels, while the exposure time to insecticides increased larval mortality rates. The two insecticides that positively affected the mortality rates were thiamethoxam (reference category) and pirimiphos-methyl (beta coefficient = 0.141; *p*-value < 0.05). The two remaining insecticides, alpha-cypermethrin and deltamethrin, and control were the less effective regarding the increase of mortality rates as indicated by the results of regression analysis.

Next, it was observed that the insect species is also significant for the prediction of the response variable. The mortality rate of *T. confusum* was significantly increased in comparison to *E. kuehniella* (beta coefficient = −0.203; *p*-value < 0.05).

The increase of dose increased the mortality rate (reference category of 0.25 mg (a.i.)/cm^2^ differs statistically from doses of both 0.05 and 0.10 mg (a.i.)/cm^2^). There were also statistically significant differences between the doses of 0.05 and 0.10 (a.i.)/cm^2^ mg, since the 95% confidence intervals of the two dosages are not overlapping (Table 3).

Significantly higher mortality rates were observed when the insecticides were applied on both surfaces (beta coefficient = 0.057; *p*-value > 0.05) or when they were applied on one surface, but insects were exposed to treated surface (reference category), than when they were applied on one surface but insects were exposed to untreated surface (beta coefficient = −0.195; *p*-value < 0.05). 

Concerning the effects of exposure time, statistical modeling analysis showed that its effect on mortality rate is not negligible. The increase of exposure time resulted in the increase of mortality rate ($\sigma_{exposure}^{2}=0.012$) in comparison to the overall variance accounted from random effects ($\sigma_{residual}^{2}=0.021$), with 36.4% of the total variance due to random terms in the model being explained by the h/days of the total exposure.

Mean mortality rates and the corresponding standard errors of *T. confusum* and *E. kuehniella* larvae in all treatments are presented in Tables S1 and S2, respectively. Furthermore, mean mortality rates of *T. confusum* and *E. kuehniella* larvae at 0.10 mg (a.i.)/cm^2^ after 10 days of exposure for each insecticide are shown in Figures S1 and S2, respectively.

## 4. Discussion

Ιn terms of the statistical modeling and treatment of such type of mortality rate response data, our analysis offers interesting suggestions for the researchers. Specifically, we propose the utilization of more suitable distributional specifications concerning regressing mortality rate with chosen covariates, such as the quasi-binomial model [52,53], which capture better the nature of proportional data than the typical linear regression equation. However, we suggest the use of the latter in comparison to its binomial alternative in case where there is evidence of over dispersion in the examined data. The quasi-binomial distribution differs from the binomial and Poisson families. This is because the dispersion parameter of the quasi-binomial distribution is not fixed at one, thus it can more adequately model the presence of overdispersion in the data [54,55,56].

Our study reveals several clear findings for the control of *E. kuehniella* and *T. confusum*. Firstly, our findings showed that the type of the tested storage bag materials did not affect the mortality rates of both species when treated with the tested insecticides. This is in accordance with Kavallieratos and Boukouvala [22] who documented that when alpha-cypermethrin, chlorfenapyr, deltamethrin, and pirimiphos-methyl applied on woven polypropylene, biaxially oriented polypropylene and kraft paper storage bags performed equally against adults and larvae of the khapra beetle, *Trogoderma granarium* Everts (Coleoptera: Dermestidae). Similarly, the fecundity of the warehouse beetle, *T. variabile* did not differ significantly when exposed either on polyethylene-to-polyethylene or on polyethylene terephthalate-polyethylene packaging materials [25]. The fact that the selected types of storage bag materials do not differentiate the performance of several insecticides may affect their supply according to the cost they are offered in case that our proposed scenarios were to be implemented.

Our results showed that the tested insecticides, insect species, doses and surface treatments had a significant effect on *E. kuehniella* and *T. confusum* larvae mortality rates when applied on storage bag materials. While all four insecticides increased the mortality rates of these species compared to control, thiamethoxam and pirimiphos-methyl showed statistically significant higher mortality rates on *E. kuehniella* and *T. confusum* compared to alpha-cypermethrin and deltamethrin. This finding is important for the selection of the most suitable insecticide subject to the management of both species. In previous studies it has been documented that thiamethoxam and pirimiphos-methyl are effective against *T. confusum* either as surface treatments or as grain protectants. For example, Saglam et al. [57] postulated that all young larvae of *T. confusum* died on concrete treated with 0.10 mg thiamethoxam/cm^2^ after 14 days of exposure. Tsaganou et al. [58] found that thiamethoxam caused 90.0% mortality rate to adults of *T. confusum* on wheat at 10 ppm 4 days post-exposure. Regarding pirimiphos-methyl, a 300 CS formulation at 4 ppm caused 98.7% mortality rate to *T. confusum* adults 21 days post-exposure on wheat [59] while a 50 EC formulation killed all *T. confusum* adults on plywood, galvanized metal and ceramic tile [60] after 14 days of exposure. Similarly, Kavallieratos et al. [19] reported that pirimiphos-methyl showed increased mortality rate on the lesser grain borer, *Rhyzopertha dominica* (F.) (Coleoptera: Bostrychidae) and the rice weevil, *Sitophilus oryzae* (L.) (Coleoptera: Curculionidae) compared to alpha-cypermethrin when applied on polypropylene bags. Nevertheless, according to the authors, both insecticides showed similar effectiveness on the larger grain borer, *Prostephanus truncatus* (Horn) (Coleoptera: Bostrychidae). This further indicates that insect species respond, in terms of mortality rate, to insecticidal treatments in different ways, an issue that is also supported by our study.

*Tribolium confusum* exhibited statistically significant higher mortality rate in comparison to *E. kuehniella*. This is an important finding considering that *T. confusum* is tolerant to various active ingredients (e.g., chlorfenapyr, deltamethrin, spinetoram, spinosad, diatomaceous earths) [3,61,62,63]. Based on former studies the order of tolerance of these two species to insecticides assumes that *T. confusum* > *E. kuehniella*. For instance, Kavallieratos et al. [44] found that *E. kehniella* suffered higher mortalities than *T. confusum* on barley, oats, peeled rice, whole rice, and wheat treated with different formulations of chlorantraniliprole at 10 ppm 14 days post-exposure. Similarly, *E. kuehniella* was less tolerant than *T. confusum* when exposed to several pyrrole derivatives on wheat under a wide spectrum of doses, temperatures and relative humidity levels [64,65]. Interestingly, Scheff and Arthur [24] documented that, even within genus, *T. confusum* was more tolerant than *T. castaneum* when these pests were exposed on deltamethrin-impregnated storage bags. Therefore, there is no specific order of tolerance between *T. confusum* and *E. kuehniella* since it may be altered according to the insecticidal treatment that is followed. More experimental work is needed on this issue. 

As expected, an increase of the dose of each of the tested insecticides resulted in an increased mortality rate of both *E. kuehniella* and *T. confusum* larvae, according to the 95% confidence intervals criterion. This was also evident for *P. truncatus*, *R. dominica* or *S. oryzae* adults and *T. granarium* adults or larvae when exposed for 1, 3 and 5 days on storage bags treated with contact insecticides [19,22]. In addition, the treatment scenario affected *E. kuehniella* and *T. confusum* larval mortality rates. Our results showed that insecticides caused significantly higher mortalities to larvae exposed on bag materials with the single treated surface in comparison to the exposure on the untreated surface of the bag materials. This is due to the fact that the examined storage bags are permeable by water, and therefore the insecticide can pass from the treated to the untreated surface [19,22]. However, the quantities of toxicants that passed to the untreated surfaces caused decreased mortality rate to the exposed larvae. In contrast, there were no significant differences in mortalities of *T. granarium* adults and larvae exposed either on treated or on untreated surfaces of these same types of bags [22]. Similar observations were obtained for *P. truncatus*, *R. dominica* and *S. oryzae* adults when they were tested on insecticide treated and untreated surfaces of woven propylene storage bags [19]. Therefore, we suggest that both surfaces of the tested storage bags should be treated with contact insecticides in order to kill *E. kuehniella* and *T. confusum* which will come in constant contact with the storage bags externally (i.e., origin from the storage environment) and internally (i.e., origin from the bagged products). Though storage bags can offer efficient control of insects that will attempt to enter the bagged products, still they cannot stop the growth of the existing insects inside bags and the concomitant infestations of these products [15,18]. The latter case is crucial because the insects will attempt to escape from the infested products and find new habitats [22]. Therefore, storage bags should be efficiently “armed” to prevent the further spread of insects. However, given that we used only portions of the bags, further investigation is needed to clarify the residual activity of our management approach.

## 5. Conclusions

We examined the efficacy of insecticide treatment as well as the impact of other explanatory variables on insect mortality rate by the use of standard and newer modeling approaches (i.e., quasi-binomial), and we found the better performance of the latter. On the basis of our findings, we present evidence that insecticide treatment of certain storage bag materials could provide value in designing programs to manage *E. kuehniella* and *T. confusum*. Moreover, we found that thiamethoxam and pirimiphos-methyl produced greater mortality with constant exposure on the tested bag fragments than alpha-cypermethrin and deltamethrin and would be more useful against these noxious species. Given that different stored-product insects exhibit different patterns of susceptibility when exposed on insecticide-treated/impregnated packaging materials [18,19,24,26,66] we expect future laboratory and field studies to evaluate the application of additional insecticides on storage bags against different species of stored-product pests and their life stages, under different environmental conditions and exposure intervals in order to gather more information towards a better understanding of their toxicity.

## Figures and Tables

**Table 1:** Technical characteristics of types of bags used in this study.

Technical Characteristics	Types of Bag Materials			
	Woven Polypropylene Bag	Biaxially Oriented Polypropylene Bag	Kraft Paper Bag (Outside White Pressed Kraft Paper)	Kraft Paper Bag (Inside Brown Extensible Kraft Paper)
Thickness	100 μm	70 μm	100 μm	100 μm
Weight	47.8 g/m^2^	27.3 g/m^2^	70 g/m^2^	70 g/m^2^
Strength	44.3 kgf (warp)/ 40.2 kgf (weft)	>10 (N/15 mm) (seal)	5.6 kN/m (MD)/ 4.1 kN/m (CD) (tensile)	6.3 kN/m (MD)/ 3.9 kN/m (CD) (tensile)

MD: machine direction; CD: cross direction.

**Table 2:** Model comparisons of the Gaussian, binomial and quasi-binomial models via *LRT*.

Model	Gaussian Model		Binomial Model		Quasi-Binomial Model	
	*D*	*p*	*D*	*p*	*D*	*p*
Model 1 (Null Model)	−16,132	----	6025.5	----	5014.8	----
Model 2 (insecticide)	−16,724	*	5873.7	*	4750.8	*
Model 3 (Model 2 + insect species)	−16,801	*	5746.4	*	4733.8	*
Model 4 (Model 3 + surface type)	−16,816	*	5742.4	n.s.	4730.5	n.s.
Model 5 (Model 4 + treatment)	−16,903	*	5699.9	*	4711.7	*
Model 6 (Model 5 + dose)	−16,971	*	5677.4	*	4698.3	*
*MSPE*	0.0212		0.0217		0.0132	

*D*: likelihood ratio statistic; *P*: *p*-value of the statistical significance of *LRT*; *: Significant at 1% level of significance; n.s.: non-significant; ----: lack of comparison.

**Table 3:** Parameter estimates of the best selected model distributional specification (quasi-binomial) (5% level of significance) along with the 95% confidence intervals.

Covariate	Estimate	95% Confidence Intervals
Intercept	1.954	(−2.043, −1.867)
Insecticide (ref. category: Thiamethoxam)		
Pirimiphos-methyl	0.141	(0.067, 0.214)
Alpha-cypermethrin	−0.078	(−0.155, −0.002)
Deltamethrin	−0.001	(−0.077, 0.074)
Control	−3.344	(−3.875, −2.889)
Insect (Ref. category: *T. confusum*)		
*E. kuehniella*	−0.203	(−0.256, −0.150)
Dose (Ref. category: 0.025 mg (a.i.)/cm^2^)		
0.05 mg (a.i.)/cm^2^	0.080	(0.014, 0.147)
0.10 mg (a.i.)/cm^2^	0.217	(0.152, 0.282)
Surface (Ref. category: one surface is treated, insects were exposed to treated surface)		
One surface is treated, insects were exposed to untreated surface	−0.195	(−0.261, −0.129)
Both surfaces were treated	0.057	(−0.006, 0.120)
Exposure (variance of random effect) $\sigma_{exposure}^{2}$	0.012	

## Data Availability

The data presented in this study are available in supplementary material.

## Supplementary Materials

The following are available online at https://www.mdpi.com/2075-4450/12/2/182/s1, Figure S1: Mean mortality rates (% ± SE) of *Tribolium confusum* larvae exposed for 10 days onto (a) woven polypropylene; (b) biaxially oriented polypropylene; (c) kraft paper storage bag materials treated with thiamethoxam, pirimiphos-methyl, alpha-cypermethrin and deltamethrin at 0.10 mg (a.i.)/cm^2^, Figure S2: Mean mortality rates (% ± SE) of *Ephestia kuehniella* larvae exposed for 10 days onto (a) woven polypropylene; (b) biaxially oriented polypropylene; (c) kraft paper storage bag materials treated with thiamethoxam, pirimiphos-methyl, alpha-cypermethrin and deltamethrin at 0.10 mg (a.i.)/cm^2^. Table S1: Mean mortality rates (% ± SE) of *Tribolium confusum* larvae exposed for 1, 2, 3 h and 1, 2, 3 and 10 days onto woven polypropylene, biaxially oriented polypropylene and kraft paper storage bag materials treated with thiamethoxam, pirimiphos-methyl, alpha-cypermethrin and deltamethrin at three doses. Control mortality rate is <5%., Table S2: Mean mortality rates (% ± SE) of *Ephestia kuehniella* larvae exposed for 1, 2, 3 h and 1, 2, 3 and 10 days onto woven polypropylene, biaxially oriented polypropylene and kraft paper storage bag materials treated with thiamethoxam, pirimiphos-methyl, alpha-cypermethrin and deltamethrin at three doses. Control mortality rate is <5%.
