# Supplementary material for: (Quasi)-Binomial vs. Gaussian Models to Evaluate Thiamethoxam, Pirimiphos-Methyl, Alpha-Cypermethrin and Deltamethrin on Different Types of Storage Bag Materials Against Ephestia kuehniella Zeller (Lepidoptera: Pyralidae) and Tribolium confusum Jacquelin du Val (Coleoptera: Tenebrionidae)
Source: Insects. 2021 Feb 21;12(2):182. doi: 10.3390/insects12020182 (PMC7926401; doi:10.3390/insects12020182)
Supplement: Supplementary file 1 [file insects-12-00182-s001.pdf]

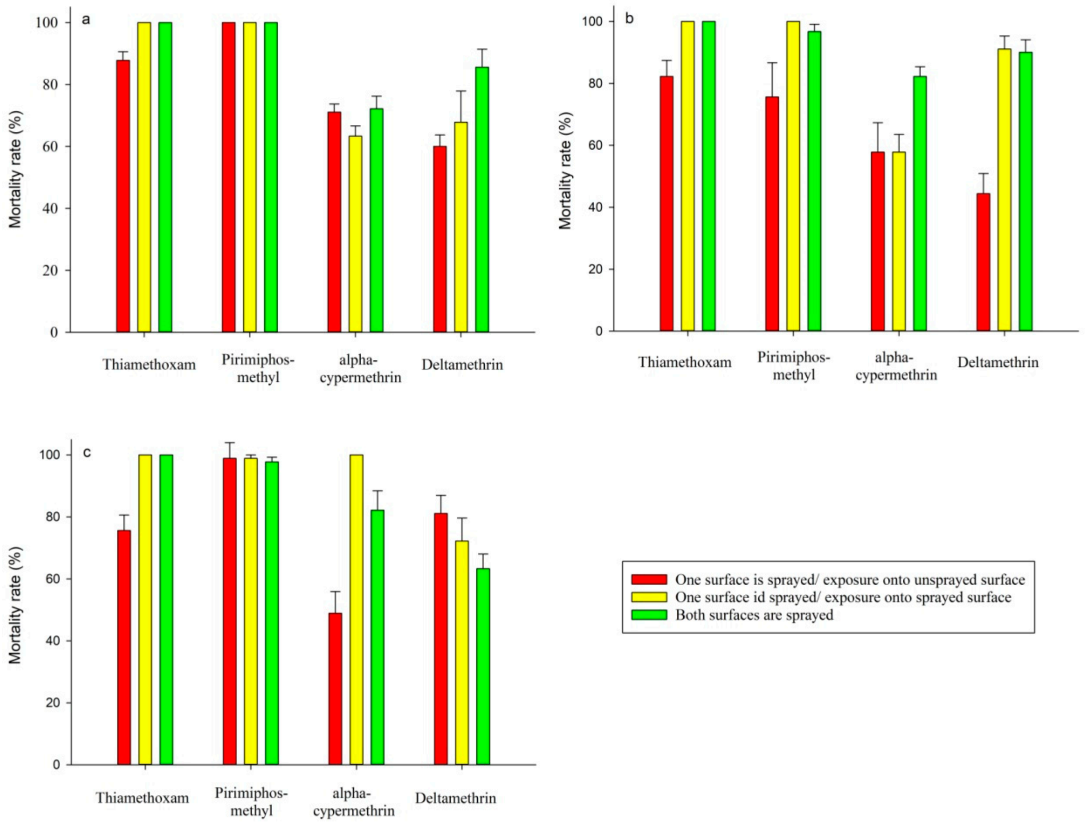

Figure S1. Mean mortality rates (% ± SE) of *Tribolium confusum* larvae exposed for 10 days onto (a) woven polypropylene; (b) biaxially oriented polypropylene; (c) kraft paper storage bag materials treated with thiamethoxam, pirimiphos-methyl, alpha-cypermethrin and deltamethrin at 0.10 mg (a.i.)/cm^2.

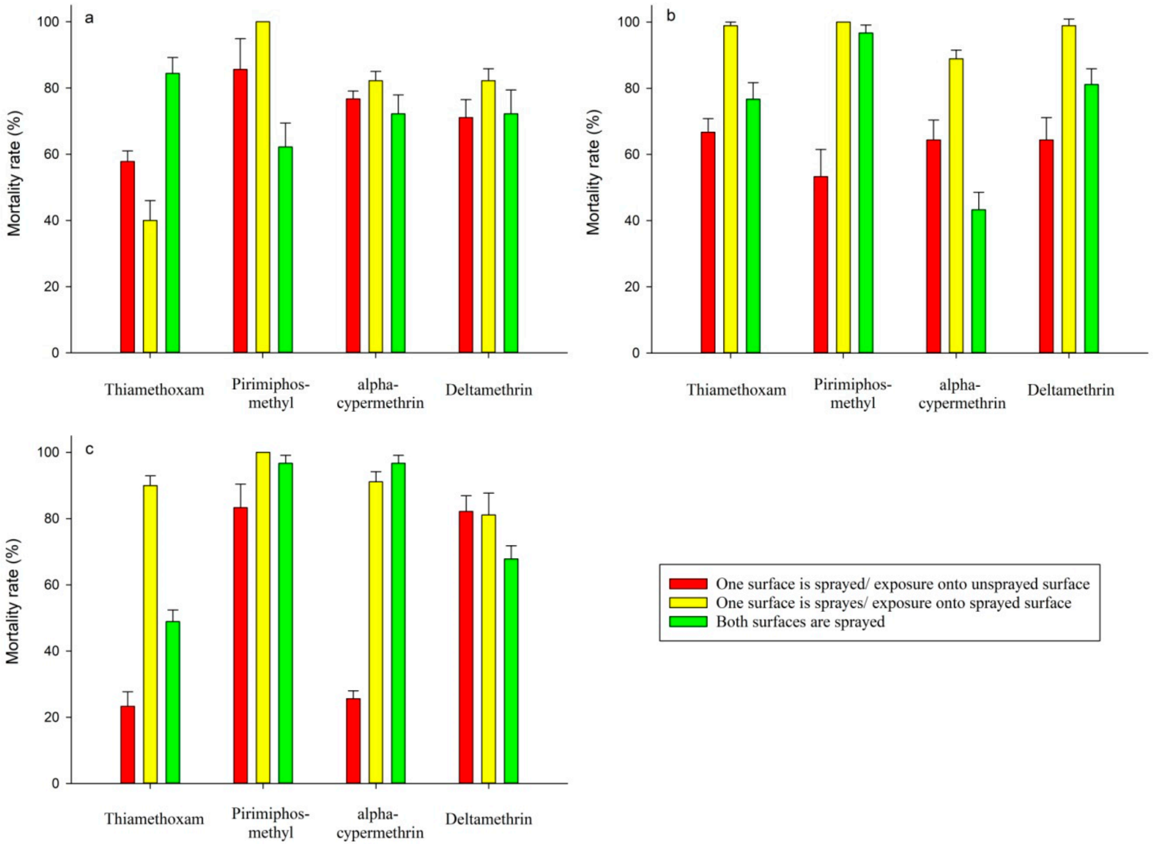

Figure S2. Mean mortality rates (% ± SE) of *Ephestia kuehniella* larvae exposed for 10 days onto (a) woven polypropylene; (b) biaxially oriented polypropylene; (c) kraft paper storage bag materials treated with thiamethoxam, pirimiphos-methyl, alpha-cypermethrin and deltamethrin at 0.10 mg (a.i.)/cm².

Table S1. Mean mortality rate (% ± SE) of *Tribolium confusum* larvae exposed for 1, 2, 3 h and 1, 2, 3 and 10 days onto WPP, BOPP or KP storage bag materials treated with thiamethoxam, pirimiphos-methyl, alpha-cypermethrin and deltamethrin in three doses. Control mortality rate is <5%.

**Bag material**	**WPP**							
**Sprayed surface**	**One surface is sprayed/ exposure onto unsprayed surface**	**Exposure**						
**Insecticide**	**Dose**	**1 h**	**2 h**	**3 h**	**1 day**	**2 days**	**3 days**	**10 days**
Thiamethoxam	0.025 mg (a.i.)/cm²	0.0±0.0	0.0±0.0	0.0±0.0	0.0±0.0	5.6±3.4	18.9±4.2	43.3±5.5
	0.05 mg (a.i.)/cm²	0.0±0.0	0.0±0.0	0.0±0.0	1.1±1.1	14.4±2.4	25.6±4.4	53.3±5.5
	0.10 mg (a.i.)/cm²	0.0±0.0	0.0±0.0	0.0±0.0	3.3±1.7	14.4±4.4	31.1±4.2	87.8±2.8
Pirimiphos-methyl	0.025 mg (a.i.)/cm²	0.0±0.0	0.0±0.0	0.0±0.0	0.0±0.0	0.0±0.0	3.3±1.7	56.7±5.8
	0.05 mg (a.i.)/cm²	0.0±0.0	0.0±0.0	0.0±0.0	1.1±1.1	3.3±2.4	7.8±3.2	92.2±5.2
	0.10 mg (a.i.)/cm²	0.0±0.0	0.0±0.0	1.1±1.1	2.2±1.5	3.3±2.4	13.3±5.5	100.0±0.0
alpha-cypermethrin	0.025 mg (a.i.)/cm²	0.0±0.0	0.0±0.0	0.0±0.0	1.1±1.1	4.4±1.8	22.2±4.7	55.6±3.8
	0.05 mg (a.i.)/cm²	0.0±0.0	0.0±0.0	0.0±0.0	3.3±3.3	13.3±4.4	27.8±7.2	63.3±2.9
	0.10 mg (a.i.)/cm²	0.0±0.0	0.0±0.0	0.0±0.0	0.0±0.0	14.4±4.1	31.1±4.8	71.1±2.6
Deltamethrin	0.025 mg (a.i.)/cm²	0.0±0.0	0.0±0.0	0.0±0.0	0.0±0.0	0.0±0.0	3.3±1.7	20.0±5.5
	0.05 mg (a.i.)/cm²	0.0±0.0	0.0±0.0	0.0±0.0	0.0±0.0	8.9±3.5	10.0±4.1	23.3±5.0
	0.10 mg (a.i.)/cm²	0.0±0.0	0.0±0.0	0.0±0.0	0.0±0.0	12.2±3.6	22.2±3.2	60.0±3.7
Sprayed surface	One surface is sprayed/ exposure onto sprayed surface							
Insecticide	Dose							
Thiamethoxam	0.025 mg (a.i.)/cm²	0.0±0.0	0.0±0.0	0.0±0.0	5.6±2.4	52.2±2.2	91.1±4.6	100.0±0.0
	0.05 mg (a.i.)/cm²	0.0±0.0	0.0±0.0	2.2±1.5	13.3±2.9	60.0±5.0	88.9±6.1	100.0±0.0
	0.10 mg (a.i.)/cm²	0.0±0.0	5.6±1.8	10.0±2.4	18.9±2.6	61.1±3.1	96.7±1.7	100.0±0.0
Pirimiphos-	0.025 mg (a.i.)/cm²	0.0±0.0	0.0±0.0	0.0±0.0	8.9±2.6	37.8±4.7	64.4±2.4	100.0±0.0

methyl								
	0.05 mg (a.i.)/cm²	0.0±0.0	0.0±0.0	0.0±0.0	10.0±3.7	47.8±3.2	100.0±0.0	100.0±0.0
	0.10 mg (a.i.)/cm²	0.0±0.0	0.0±0.0	0.0±0.0	11.1±3.5	61.1±2.6	100.0±0.0	100.0±0.0
alpha-cypermethrin	0.025 mg (a.i.)/cm²	0.0±0.0	0.0±0.0	0.0±0.0	3.3±1.7	11.1±3.5	15.6±4.8	28.9±3.9
	0.05 mg (a.i.)/cm²	0.0±0.0	0.0±0.0	0.0±0.0	7.8±2.8	24.4±3.8	31.1±4.6	46.7±5.8
	0.10 mg (a.i.)/cm²	0.0±0.0	0.0±0.0	0.0±0.0	13.3±3.7	27.8±5.5	41.1±4.6	63.3±3.3
Deltamethrin	0.025 mg (a.i.)/cm²	0.0±0.0	0.0±0.0	0.0±0.0	0.0±0.0	0.0±0.0	4.4±2.9	14.4±4.1
	0.05 mg (a.i.)/cm²	0.0±0.0	0.0±0.0	0.0±0.0	1.1±1.1	7.8±3.2	15.6±3.8	35.6±4.4
	0.10 mg (a.i.)/cm²	0.0±0.0	0.0±0.0	0.0±0.0	4.4±2.9	21.1±3.1	50.0±11.4	67.8±10.1
Sprayed surface	Both surfaces are sprayed							
Insecticide	Dose							
Thiamethoxam	0.025 mg (a.i.)/cm²	0.0±0.0	0.0±0.0	0.0±0.0	4.4±2.4	11.1±2.6	23.3±2.4	90.0±2.9
	0.05 mg (a.i.)/cm²	0.0±0.0	0.0±0.0	0.0±0.0	0.0±0.0	13.3±2.9	28.9±4.2	97.8±1.5
	0.10 mg (a.i.)/cm²	0.0±0.0	0.0±0.0	0.0±0.0	10.0±2.4	15.6±5.0	31.1±5.6	100.0±0.0
Pirimiphos-methyl	0.025 mg (a.i.)/cm²	0.0±0.0	0.0±0.0	0.0±0.0	1.1±1.1	2.2±2.2	48.9±8.9	67.8±14.4
	0.05 mg (a.i.)/cm²	0.0±0.0	0.0±0.0	0.0±0.0	2.2±2.2	15.6±4.4	63.3±6.0	96.7±4.7
	0.10 mg (a.i.)/cm²	0.0±0.0	0.0±0.0	0.0±0.0	4.4±2.4	45.6±3.8	73.3±6.0	100.0±0.0
alpha-cypermethrin	0.025 mg (a.i.)/cm²	0.0±0.0	0.0±0.0	0.0±0.0	0.0±0.0	1.1±1.1	8.9±2.0	54.4±3.8
	0.05 mg (a.i.)/cm²	0.0±0.0	0.0±0.0	0.0±0.0	1.1±1.1	4.4±1.8	12.2±3.2	56.7±2.4
	0.10 mg (a.i.)/cm²	0.0±0.0	0.0±0.0	0.0±0.0	2.2±1.5	8.9±2.6	21.1±4.8	72.2±4.0
Deltamethrin	0.025 mg (a.i.)/cm²	0.0±0.0	0.0±0.0	2.2±1.5	6.7±1.7	22.2±4.0	43.3±8.8	80.0±5.5
	0.05 mg (a.i.)/cm²	0.0±0.0	0.0±0.0	7.8±2.2	20.0±6.9	33.3±6.7	48.9±8.2	81.1±4.2
	0.10 mg (a.i.)/cm²	0.0±0.0	0.0±0.0	12.2±4.0	22.2±4.0	37.8±5.7	51.1±5.1	85.6±5.8
Bag material	BOPP							
Sprayed surface	One surface is sprayed/ exposure onto unsprayed surface							

Insecticide	Dose							
Thiamethoxam	0.025 mg (a.i.)/cm²	0.0±0.0	0.0±0.0	0.0±0.0	0.0±0.0	11.1±3.1	25.6±3.8	62.2±6.2
	0.05 mg (a.i.)/cm²	0.0±0.0	0.0±0.0	0.0±0.0	1.1±1.1	13.3±3.3	37.8±5.5	77.8±7.0
	0.10 mg (a.i.)/cm²	0.0±0.0	0.0±0.0	0.0±0.0	3.3±1.7	17.8±4.7	40.0±2.9	82.2±5.2
Pirimiphos-methyl	0.025 mg (a.i.)/cm²	0.0±0.0	0.0±0.0	0.0±0.0	0.0±0.0	0.0±0.0	11.1±5.1	28.9±3.5
	0.05 mg (a.i.)/cm²	0.0±0.0	0.0±0.0	0.0±0.0	0.0±0.0	2.2±1.5	11.1±3.5	41.1±11.1
	0.10 mg (a.i.)/cm²	0.0±0.0	0.0±0.0	0.0±0.0	4.4±1.8	16.7±2.9	51.1±10.7	75.6±11.1
alpha-cypermethrin	0.025 mg (a.i.)/cm²	0.0±0.0	0.0±0.0	0.0±0.0	0.0±0.0	3.3±1.7	7.8±3.2	28.9±6.6
	0.05 mg (a.i.)/cm²	0.0±0.0	0.0±0.0	0.0±0.0	0.0±0.0	3.3±2.4	7.8±4.7	48.9±7.0
	0.10 mg (a.i.)/cm²	0.0±0.0	0.0±0.0	0.0±0.0	1.1±1.1	4.4±3.4	12.2±4.3	57.8±9.5
Deltamethrin	0.025 mg (a.i.)/cm²	0.0±0.0	0.0±0.0	0.0±0.0	0.0±0.0	0.0±0.0	6.7±5.5	14.4±5.6
	0.05 mg (a.i.)/cm²	0.0±0.0	0.0±0.0	0.0±0.0	0.0±0.0	3.3±2.4	20.0±7.5	32.2±6.2
	0.10 mg (a.i.)/cm²	5.6±5.6	5.6±5.6	5.6±5.6	5.6±5.6	5.6±5.6	21.1±3.5	44.4±6.5
Sprayed surface	One surface is sprayed/ exposure onto sprayed surface							
Insecticide	Dose							
Thiamethoxam	0.025 mg (a.i.)/cm²	1.1±1.1	1.1±1.1	1.1±1.1	11.1±3.5	51.1±8.7	67.8±8.9	100.0±0.0
	0.05 mg (a.i.)/cm²	1.1±1.1	2.2±1.5	2.2±1.5	13.3±4.4	43.3±7.5	76.7±5.3	100.0±0.0
	0.10 mg (a.i.)/cm²	4.4±1.8	5.6±1.8	5.6±1.8	22.2±4.5	56.7±6.7	85.6±1.8	100.0±0.0
Pirimiphos-methyl	0.025 mg (a.i.)/cm²	0.0±0.0	0.0±0.0	0.0±0.0	3.3±2.4	28.9±4.2	56.7±4.7	100.0±0.0
	0.05 mg (a.i.)/cm²	0.0±0.0	0.0±0.0	0.0±0.0	7.8±3.6	45.6±4.8	100.0±0.0	100.0±0.0
	0.10 mg (a.i.)/cm²	0.0±0.0	0.0±0.0	0.0±0.0	15.6±3.4	55.6±2.4	100.0±0.0	100.0±0.0
alpha-cypermethrin	0.025 mg (a.i.)/cm²	0.0±0.0	0.0±0.0	0.0±0.0	3.3±1.7	6.7±2.9	12.2±3.6	30.0±3.7
	0.05 mg (a.i.)/cm²	0.0±0.0	0.0±0.0	0.0±0.0	5.6±2.4	11.1±3.5	21.1±5.6	45.6±5.8
	0.10 mg (a.i.)/cm²	0.0±0.0	0.0±0.0	0.0±0.0	5.6±2.4	11.1±3.1	28.9±3.5	57.8±5.7
Deltamethrin	0.025 mg (a.i.)/cm²	0.0±0.0	0.0±0.0	0.0±0.0	1.1±1.1	5.6±2.4	15.6±4.1	66.7±4.7

	0.05 mg (a.i.)/cm²	0.0±0.0	0.0±0.0	1.1±1.1	1.1±1.1	6.7±3.3	16.7±4.1	74.4±5.8
	0.10 mg (a.i.)/cm²	1.1±1.1	1.1±1.1	1.1±1.1	3.3±2.4	7.8±3.6	30.0±5.0	91.1±4.2
Sprayed surface	Both surfaces are sprayed							
Insecticide	Dose							
Thiamethoxam	0.025 mg (a.i.)/cm²	0.0±0.0	0.0±0.0	0.0±0.0	3.3±1.7	3.3±1.7	12.2±1.5	97.8±1.5
	0.05 mg (a.i.)/cm²	0.0±0.0	0.0±0.0	0.0±0.0	11.1±5.4	14.4±5.0	28.9±5.9	100.0±0.0
	0.10 mg (a.i.)/cm²	0.0±0.0	0.0±0.0	0.0±0.0	15.6±5.0	23.3±6.2	61.1±7.5	100.0±0.0
Pirimiphos-methyl	0.025 mg (a.i.)/cm²	0.0±0.0	0.0±0.0	0.0±0.0	1.1±1.1	8.9±3.1	12.2±3.6	87.8±3.6
	0.05 mg (a.i.)/cm²	0.0±0.0	0.0±0.0	0.0±0.0	4.4±1.8	10.0±2.9	21.1±3.9	94.4±2.4
	0.10 mg (a.i.)/cm²	0.0±0.0	0.0±0.0	0.0±0.0	5.6±2.9	15.6±4.4	34.4±6.0	96.7±2.4
alpha-cypermethrin	0.025 mg (a.i.)/cm²	0.0±0.0	0.0±0.0	0.0±0.0	1.1±1.1	6.7±1.7	8.9±2.6	62.2±4.0
	0.05 mg (a.i.)/cm²	0.0±0.0	0.0±0.0	0.0±0.0	0.0±0.0	7.8±2.2	16.7±4.4	72.2±5.2
	0.10 mg (a.i.)/cm²	0.0±0.0	0.0±0.0	0.0±0.0	1.1±1.1	15.6±4.1	22.2±3.6	82.2±3.2
Deltamethrin	0.025 mg (a.i.)/cm²	0.0±0.0	0.0±0.0	0.0±0.0	2.2±2.2	23.3±4.7	34.4±7.1	87.8±5.2
	0.05 mg (a.i.)/cm²	0.0±0.0	0.0±0.0	0.0±0.0	8.9±3.1	25.6±4.8	48.9±5.9	88.9±3.5
	0.10 mg (a.i.)/cm²	0.0±0.0	0.0±0.0	0.0±0.0	11.1±2.0	33.3±3.7	50.0±5.5	90.0±4.1
Bag material	KP							
Sprayed surface	One surface is sprayed/ exposure onto unsprayed surface							
Insecticide	Dose							
Thiamethoxam	0.025 mg (a.i.)/cm²	0.0±0.0	0.0±0.0	0.0±0.0	0.0±0.0	6.7±2.4	31.1±5.4	56.7±6.2
	0.05 mg (a.i.)/cm²	0.0±0.0	0.0±0.0	0.0±0.0	0.0±0.0	10.0±2.9	30.0±4.7	58.9±4.8
	0.10 mg (a.i.)/cm²	0.0±0.0	0.0±0.0	0.0±0.0	0.0±0.0	15.6±4.1	52.2±4.0	75.6±5.0
Pirimiphos-methyl	0.025 mg (a.i.)/cm²	0.0±0.0	0.0±0.0	0.0±0.0	4.4±1.8	16.7±3.7	43.3±5.8	66.7±7.8
	0.05 mg (a.i.)/cm²	0.0±0.0	0.0±0.0	0.0±0.0	4.4±1.8	20.0±3.3	75.6±6.9	90.0±5.3
	0.10 mg (a.i.)/cm²	0.0±0.0	0.0±0.0	0.0±0.0	6.7±2.4	33.3±5.8	77.8±7.8	98.9±5.1

alpha-cypermethrin	0.025 mg (a.i.)/cm^2	0.0±0.0	0.0±0.0	0.0±0.0	1.1±1.1	3.3±1.7	14.4±4.1	34.4±5.0
	0.05 mg (a.i.)/cm^2	0.0±0.0	0.0±0.0	0.0±0.0	1.1±1.1	4.4±2.9	20.0±3.3	46.7±8.5
	0.10 mg (a.i.)/cm^2	0.0±0.0	0.0±0.0	0.0±0.0	3.3±1.7	5.6±2.4	20.0±5.3	48.9±7.0
Deltamethrin	0.025 mg (a.i.)/cm^2	0.0±0.0	0.0±0.0	0.0±0.0	0.0±0.0	7.8±4.0	20.0±6.5	36.7±8.8
	0.05 mg (a.i.)/cm^2	0.0±0.0	0.0±0.0	0.0±0.0	0.0±0.0	15.6±5.0	28.9±7.4	52.2±5.7
	0.10 mg (a.i.)/cm^2	3.3±3.3	3.3±3.3	3.3±3.3	3.3±3.3	18.9±3.1	34.4±6.3	81.1±5.9
Sprayed surface	One surface is sprayed/ exposure onto sprayed surface							
Insecticide	Dose							
Thiamethoxam	0.025 mg (a.i.)/cm^2	0.0±0.0	0.0±0.0	0.0±0.0	0.0±0.0	46.7±3.3	73.3±6.7	97.8±1.5
	0.05 mg (a.i.)/cm^2	0.0±0.0	0.0±0.0	2.2±2.2	14.4±2.4	52.2±6.4	94.4±3.8	98.9±1.1
	0.10 mg (a.i.)/cm^2	0.0±0.0	0.0±0.0	0.0±0.0	15.6±2.4	75.6±4.1	96.7±2.4	100.0±0.0
Pirimiphos-methyl	0.025 mg (a.i.)/cm^2	0.0±0.0	0.0±0.0	0.0±0.0	18.9±2.0	57.8±3.2	84.4±4.8	94.4±2.9
	0.05 mg (a.i.)/cm^2	0.0±0.0	0.0±0.0	0.0±0.0	24.4±4.4	60.0±4.7	92.2±5.2	95.6±4.4
	0.10 mg (a.i.)/cm^2	0.0±0.0	0.0±0.0	0.0±0.0	31.1±2.0	61.1±4.8	97.8±1.5	98.9±1.1
alpha-cypermethrin	0.025 mg (a.i.)/cm^2	0.0±0.0	0.0±0.0	0.0±0.0	5.6±2.4	28.9±5.4	57.8±4.0	82.2±3.2
	0.05 mg (a.i.)/cm^2	1.1±1.1	1.1±1.1	1.1±1.1	8.9±3.5	30.0±4.7	71.1±4.6	87.8±3.2
	0.10 mg (a.i.)/cm^2	2.2±1.5	2.2±1.5	3.3±1.7	20.0±3.3	50.0±3.7	98.9±1.1	100.0±0.0
Deltamethrin	0.025 mg (a.i.)/cm^2	0.0±0.0	1.1±1.1	1.1±1.1	1.1±1.1	5.6±3.4	10.0±3.7	42.2±6.8
	0.05 mg (a.i.)/cm^2	1.1±1.1	1.1±1.1	2.2±1.5	2.2±1.5	8.9±3.5	11.1±5.9	63.3±8.2
	0.10 mg (a.i.)/cm^2	8.9±7.7	10.0±7.6	10.0±7.6	10.0±7.6	10.0±7.6	21.1±7.0	72.2±7.4
Sprayed surface	Both surfaces are sprayed							
Insecticide	Dose							
Thiamethoxam	0.025 mg (a.i.)/cm^2	0.0±0.0	0.0±0.0	0.0±0.0	0.0±0.0	52.2±4.0	81.1±3.9	93.3±2.9
	0.05 mg (a.i.)/cm^2	0.0±0.0	0.0±0.0	0.0±0.0	0.0±0.0	54.4±4.4	80.0±3.3	96.7±1.7
	0.10 mg (a.i.)/cm^2	0.0±0.0	0.0±0.0	0.0±0.0	4.4±2.4	68.9±2.6	100.0±0.0	100.0±0.0
Pirimiphos-	0.025 mg (a.i.)/cm^2	0.0±0.0	0.0±0.0	0.0±0.0	0.0±0.0	7.8±2.2	47.8±5.2	86.7±2.9

methyl								
	0.05 mg (a.i.)/cm^2	0.0±0.0	0.0±0.0	0.0±0.0	1.1±1.1	7.8±2.2	52.2±8.1	94.4±2.4
	0.10 mg (a.i.)/cm^2	0.0±0.0	0.0±0.0	0.0±0.0	3.3±1.7	15.6±3.8	62.2±5.7	97.8±1.5
alpha-cypermethrin	0.025 mg (a.i.)/cm^2	0.0±0.0	0.0±0.0	0.0±0.0	0.0±0.0	2.2±1.5	7.8±2.8	63.3±2.9
	0.05 mg (a.i.)/cm^2	0.0±0.0	0.0±0.0	0.0±0.0	0.0±0.0	3.3±1.7	8.9±3.5	65.6±5.3
	0.10 mg (a.i.)/cm^2	0.0±0.0	0.0±0.0	0.0±0.0	0.0±0.0	4.4±1.8	22.2±5.7	82.2±6.2
Deltamethrin	0.025 mg (a.i.)/cm^2	0.0±0.0	0.0±0.0	0.0±0.0	5.6±1.8	12.2±2.8	20.0±3.7	50.0±8.5
	0.05 mg (a.i.)/cm^2	0.0±0.0	0.0±0.0	0.0±0.0	10.0±3.7	13.3±3.3	23.3±2.4	52.2±6.0
	0.10 mg (a.i.)/cm^2	0.0±0.0	0.0±0.0	0.0±0.0	12.2±4.3	17.8±4.7	28.9±6.1	63.3±4.7

Table S2. Mean mortality rate (% ± SE) of *Ephestia kuehniella* larvae exposed for 1, 2, 3 h and 1, 2, 3 and 10 days onto WPP, BOPP or KP storage bag materials treated with thiamethoxam, pirimiphos-methyl, alpha-cypermethrin and deltamethrin in three doses. Control mortality rate is <5%.

**Bag material**	**WPP**							
**Sprayed surface**	**One surface is sprayed/ exposure onto unsprayed surface**	**Exposure**						
**Insecticide**	**Dose**	**1 h**	**2 h**	**3 h**	**1 day**	**2 days**	**3 days**	**10 days**
Thiamethoxam	0.025 mg (a.i.)/cm^2	0.0±0.0	0.0±0.0	0.0±0.0	0.0±0.0	1.1±1.1	10.0±3.3	31.1±2.6
	0.05 mg (a.i.)/cm^2	0.0±0.0	0.0±0.0	0.0±0.0	0.0±0.0	2.3±1.7	11.1±3.5	40.0±3.3
	0.10 mg (a.i.)/cm^2	0.0±0.0	0.0±0.0	0.0±0.0	2.2±1.5	5.6±2.4	15.6±3.8	57.8±3.2
Pirimiphos-methyl	0.025 mg (a.i.)/cm^2	0.0±0.0	0.0±0.0	0.0±0.0	2.2±2.2	5.6±1.8	14.4±4.4	56.7±8.3
	0.05 mg (a.i.)/cm^2	0.0±0.0	0.0±0.0	0.0±0.0	5.6±1.8	5.6±4.4	26.7±7.5	68.9±9.6
	0.10 mg (a.i.)/cm^2	0.0±0.0	0.0±0.0	0.0±0.0	5.6±2.4	11.1±4.6	23.3±6.5	85.6±9.3
alpha-cypermethrin	0.025 mg (a.i.)/cm^2	0.0±0.0	0.0±0.0	0.0±0.0	1.1±1.1	12.2±3.2	26.7±4.1	60.0±4.4
	0.05 mg (a.i.)/cm^2	0.0±0.0	0.0±0.0	0.0±0.0	2.2±1.5	13.3±3.3	27.8±4.9	62.2±2.8

	0.10 mg (a.i.)/cm²	0.0±0.0	0.0±0.0	0.0±0.0	4.4±1.8	18.9±3.1	37.8±3.6	76.7±2.4
Deltamethrin	0.025 mg (a.i.)/cm²	0.0±0.0	0.0±0.0	8.9±3.1	20.0±2.9	28.9±3.9	31.1±4.5	53.3±5.3
	0.05 mg (a.i.)/cm²	0.0±0.0	0.0±0.0	8.9±2.6	23.3±3.3	34.4±3.4	42.2±6.2	64.4±5.6
	0.10 mg (a.i.)/cm²	0.0±0.0	0.0±0.0	11.1±2.6	24.4±2.9	37.8±2.2	47.8±3.6	71.1±5.4
Sprayed surface	One surface is sprayed/ exposure onto sprayed surface							
Insecticide	Dose							
Thiamethoxam	0.025 mg (a.i.)/cm²	0.0±0.0	0.0±0.0	0.0±0.0	4.4±1.8	7.8±2.2	16.7±3.7	18.9±3.1
	0.05 mg (a.i.)/cm²	0.0±0.0	0.0±0.0	0.0±0.0	7.8±2.2	20.0±4.7	28.9±5.9	40.0±3.3
	0.10 mg (a.i.)/cm²	0.0±0.0	0.0±0.0	0.0±0.0	13.3±2.4	25.6±3.4	37.8±4.0	40.0±6.0
Pirimiphos-methyl	0.025 mg (a.i.)/cm²	0.0±0.0	0.0±0.0	0.0±0.0	5.6±2.4	15.6±2.9	50.0±4.1	100.0±0.0
	0.05 mg (a.i.)/cm²	0.0±0.0	0.0±0.0	0.0±0.0	10.0±3.7	43.3±4.1	100.0±0.0	100.0±0.0
	0.10 mg (a.i.)/cm²	0.0±0.0	0.0±0.0	0.0±0.0	13.3±4.1	44.4±2.9	100.0±0.0	100.0±0.0
alpha-cypermethrin	0.025 mg (a.i.)/cm²	0.0±0.0	0.0±0.0	0.0±0.0	24.4±2.4	34.4±3.4	43.3±4.1	65.6±3.8
	0.05 mg (a.i.)/cm²	0.0±0.0	0.0±0.0	2.2±2.2	31.1±3.1	38.9±3.9	50.0±4.7	73.3±2.4
	0.10 mg (a.i.)/cm²	3.3±1.7	3.3±1.7	3.3±1.7	37.8±2.2	54.4±4.1	63.3±3.3	82.2±2.8
Deltamethrin	0.025 mg (a.i.)/cm²	0.0±0.0	0.0±0.0	0.0±0.0	4.4±1.8	37.8±5.7	56.7±5.0	70.0±3.7
	0.05 mg (a.i.)/cm²	0.0±0.0	0.0±0.0	0.0±0.0	11.1±3.1	26.7±6.5	53.3±5.3	61.1±3.9
	0.10 mg (a.i.)/cm²	0.0±0.0	0.0±0.0	0.0±0.0	20.0±5.8	31.1±8.6	76.7±3.7	82.2±3.6
Sprayed surface	Both surfaces are sprayed							
Insecticide	Dose							
Thiamethoxam	0.025 mg (a.i.)/cm²	0.0±0.0	0.0±0.0	0.0±0.0	5.6±2.9	23.3±5.3	44.4±2.9	61.1±2.6
	0.05 mg (a.i.)/cm²	0.0±0.0	0.0±0.0	0.0±0.0	8.9±2.0	22.2±4.9	44.4±6.0	65.6±4.4
	0.10 mg (a.i.)/cm²	0.0±0.0	0.0±0.0	0.0±0.0	8.9±2.6	24.4±3.8	45.6±5.3	84.4±4.8
Pirimiphos-methyl	0.025 mg (a.i.)/cm²	0.0±0.0	0.0±0.0	0.0±0.0	3.3±2.4	8.9±4.6	11.1±4.6	38.9±6.6
	0.05 mg (a.i.)/cm²	0.0±0.0	0.0±0.0	0.0±0.0	5.6±3.4	14.4±3.4	23.3±5.3	60.0±6.7
	0.10 mg (a.i.)/cm²	0.0±0.0	0.0±0.0	0.0±0.0	7.8±3.6	14.4±5.3	20.0±5.5	62.2±7.2

alpha-cypermethrin	0.025 mg (a.i.)/cm²	0.0±0.0	0.0±0.0	0.0±0.0	5.6±2.4	21.1±4.2	35.6±4.4	56.7±3.3
	0.05 mg (a.i.)/cm²	0.0±0.0	0.0±0.0	0.0±0.0	6.7±2.9	25.6±3.4	38.9±4.2	64.4±2.4
	0.10 mg (a.i.)/cm²	0.0±0.0	0.0±0.0	0.0±0.0	4.4±1.8	28.9±5.4	40.0±4.7	72.2±5.7
Deltamethrin	0.025 mg (a.i.)/cm²	0.0±0.0	0.0±0.0	1.1±1.1	7.8±2.8	18.9±3.5	36.7±3.3	56.7±8.7
	0.05 mg (a.i.)/cm²	0.0±0.0	0.0±0.0	2.2±1.5	8.9±2.6	21.1±3.5	36.7±3.3	61.1±4.6
	0.10 mg (a.i.)/cm²	0.0±0.0	0.0±0.0	2.2±2.2	11.1±2.0	24.4±3.4	36.7±5.5	72.2±7.2
Bag material	BOPP							
Sprayed surface	One surface is sprayed/ exposure onto unsprayed surface							
Insecticide	Dose							
Thiamethoxam	0.025 mg (a.i.)/cm²	0.0±0.0	0.0±0.0	0.0±0.0	0.0±0.0	2.2±2.2	11.1±2.6	41.1±4.6
	0.05 mg (a.i.)/cm²	0.0±0.0	0.0±0.0	0.0±0.0	1.1±1.1	4.4±1.8	17.8±5.7	58.9±3.1
	0.10 mg (a.i.)/cm²	0.0±0.0	0.0±0.0	0.0±0.0	4.4±1.8	10.0±3.7	25.6±5.6	66.7±4.1
Pirimiphos-methyl	0.025 mg (a.i.)/cm²	0.0±0.0	0.0±0.0	0.0±0.0	0.0±0.0	4.4±2.4	8.9±3.5	34.4±6.0
	0.05 mg (a.i.)/cm²	0.0±0.0	0.0±0.0	0.0±0.0	2.2±1.5	6.7±2.9	15.6±6.3	44.4±6.7
	0.10 mg (a.i.)/cm²	0.0±0.0	0.0±0.0	0.0±0.0	3.3±2.4	8.9±4.6	22.2±6.8	53.3±8.2
alpha-cypermethrin	0.025 mg (a.i.)/cm²	0.0±0.0	0.0±0.0	0.0±0.0	5.6±1.8	13.3±4.7	21.1±4.6	53.3±3.7
	0.05 mg (a.i.)/cm²	0.0±0.0	0.0±0.0	0.0±0.0	7.8±2.2	14.4±4.4	25.6±6.0	60.0±3.7
	0.10 mg (a.i.)/cm²	0.0±0.0	0.0±0.0	0.0±0.0	8.9±3.5	16.7±3.7	26.7±5.8	64.4±6.0
Deltamethrin	0.025 mg (a.i.)/cm²	0.0±0.0	0.0±0.0	2.2±1.5	15.6±2.4	22.2±4.3	24.4±4.4	44.4±5.0
	0.05 mg (a.i.)/cm²	4.4±3.4	6.7±3.7	16.7±5.5	26.7±7.1	37.8±6.2	43.3±6.7	63.3±9.9
	0.10 mg (a.i.)/cm²	4.4±3.4	7.8±4.5	20.0±6.2	34.4±8.7	43.3±10.8	47.8±10.9	64.4±6.7
Sprayed surface	One surface is sprayed/ exposure onto sprayed surface							
Insecticide	Dose							
Thiamethoxam	0.025 mg (a.i.)/cm²	0.0±0.0	0.0±0.0	0.0±0.0	17.8±2.2	50.0±3.3	72.2±4.0	90.0±4.1

	0.05 mg (a.i.)/cm²	0.0±0.0	0.0±0.0	0.0±0.0	23.3±1.7	63.3±2.4	82.2±3.2	93.3±2.9
	0.10 mg (a.i.)/cm²	0.0±0.0	2.2±1.5	4.4±1.8	25.6±2.9	84.4±3.8	97.8±1.5	98.9±1.1
Pirimiphos-methyl	0.025 mg (a.i.)/cm²	0.0±0.0	0.0±0.0	0.0±0.0	20.0±6.0	37.8±4.3	52.2±6.0	81.1±4.2
	0.05 mg (a.i.)/cm²	0.0±0.0	0.0±0.0	1.1±1.1	15.6±3.8	37.8±2.2	80.0±7.5	92.2±3.8
	0.10 mg (a.i.)/cm²	0.0±0.0	0.0±0.0	6.7±3.7	34.4±6.9	61.1±2.0	97.8±2.2	100.0±0.0
alpha-cypermethrin	0.025 mg (a.i.)/cm²	0.0±0.0	0.0±0.0	0.0±0.0	2.2±1.5	35.6±4.1	46.7±5.3	74.4±4.4
	0.05 mg (a.i.)/cm²	0.0±0.0	0.0±0.0	0.0±0.0	3.3±1.7	38.9±3.1	54.4±3.8	76.7±3.3
	0.10 mg (a.i.)/cm²	0.0±0.0	0.0±0.0	0.0±0.0	12.2±2.8	47.8±2.8	66.7±3.7	88.9±2.6
Deltamethrin	0.025 mg (a.i.)/cm²	0.0±0.0	0.0±0.0	1.1±1.1	8.9±3.5	24.4±4.8	41.1±6.8	86.7±2.9
	0.05 mg (a.i.)/cm²	0.0±0.0	0.0±0.0	7.8±2.8	22.2±6.2	27.8±2.8	48.9±7.4	95.6±1.8
	0.10 mg (a.i.)/cm²	0.0±0.0	0.0±0.0	10.0±3.7	22.2±3.2	32.2±7.2	53.3±9.4	98.9±2.0
Sprayed surface	Both surfaces are sprayed							
Insecticide	Dose							
Thiamethoxam	0.025 mg (a.i.)/cm²	0.0±0.0	0.0±0.0	0.0±0.0	5.6±2.4	12.2±4.0	27.8±4.3	47.8±3.2
	0.05 mg (a.i.)/cm²	0.0±0.0	0.0±0.0	0.0±0.0	6.7±1.7	18.9±2.6	42.2±4.7	64.4±3.8
	0.10 mg (a.i.)/cm²	0.0±0.0	0.0±0.0	0.0±0.0	15.6±3.8	30.0±4.4	46.7±5.0	76.7±5.0
Pirimiphos-methyl	0.025 mg (a.i.)/cm²	0.0±0.0	0.0±0.0	0.0±0.0	0.0±0.0	10.0±2.9	20.0±5.3	45.6±9.7
	0.05 mg (a.i.)/cm²	0.0±0.0	0.0±0.0	0.0±0.0	0.0±0.0	18.9±5.4	40.0±8.0	58.9±7.7
	0.10 mg (a.i.)/cm²	0.0±0.0	0.0±0.0	0.0±0.0	0.0±0.0	40.0±4.7	65.6±7.1	96.7±2.4
alpha-cypermethrin	0.025 mg (a.i.)/cm²	0.0±0.0	0.0±0.0	0.0±0.0	2.2±2.2	13.3±4.1	18.9±6.1	28.9±4.8
	0.05 mg (a.i.)/cm²	0.0±0.0	0.0±0.0	0.0±0.0	3.3±2.4	13.3±5.0	21.1±4.6	34.4±8.5
	0.10 mg (a.i.)/cm²	0.0±0.0	0.0±0.0	0.0±0.0	4.4±1.8	14.4±2.9	25.6±5.0	43.3±5.3
Deltamethrin	0.025 mg (a.i.)/cm²	0.0±0.0	0.0±0.0	0.0±0.0	3.3±2.4	8.9±3.9	18.9±4.8	42.2±7.0
	0.05 mg (a.i.)/cm²	0.0±0.0	0.0±0.0	1.1±1.1	6.7±2.9	26.7±6.2	47.8±8.5	72.2±8.6
	0.10 mg (a.i.)/cm²	0.0±0.0	0.0±0.0	0.0±0.0	6.7±2.9	16.7±5.3	45.6±5.0	81.1±4.8
Bag material	KP							

Sprayed surface	One surface is sprayed/ exposure onto unsprayed surface							
Insecticide	Dose							
Thiamethoxam	0.025 mg (a.i.)/cm^2	0.0±0.0	0.0±0.0	0.0±0.0	1.1±1.1	3.3±1.7	5.6±2.4	5.6±2.1
	0.05 mg (a.i.)/cm^2	0.0±0.0	0.0±0.0	0.0±0.0	3.3±1.7	5.6±3.4	7.8±4.3	10.0±4.7
	0.10 mg (a.i.)/cm^2	0.0±0.0	0.0±0.0	0.0±0.0	4.4±1.8	8.9±2.6	12.2±2.8	23.3±4.4
Pirimiphos-methyl	0.025 mg (a.i.)/cm^2	0.0±0.0	0.0±0.0	0.0±0.0	2.2±1.5	2.2±1.5	16.7±3.4	32.2±7.2
	0.05 mg (a.i.)/cm^2	0.0±0.0	0.0±0.0	0.0±0.0	3.3±1.7	4.4±1.8	32.2±7.2	40.0±4.1
	0.10 mg (a.i.)/cm^2	0.0±0.0	0.0±0.0	0.0±0.0	3.3±1.7	8.9±2.6	33.3±3.7	83.3±7.1
alpha-cypermethrin	0.025 mg (a.i.)/cm^2	0.0±0.0	0.0±0.0	0.0±0.0	1.1±1.1	1.1±1.1	5.6±2.9	15.6±3.8
	0.05 mg (a.i.)/cm^2	0.0±0.0	0.0±0.0	0.0±0.0	1.1±1.1	5.6±2.4	8.9±3.1	18.9±3.9
	0.10 mg (a.i.)/cm^2	0.0±0.0	0.0±0.0	0.0±0.0	4.4±2.9	11.1±3.5	14.4±3.8	25.6±2.4
Deltamethrin	0.025 mg (a.i.)/cm^2	0.0±0.0	0.0±0.0	6.7±2.4	6.7±3.3	6.7±3.3	12.2±3.2	67.8±4.7
	0.05 mg (a.i.)/cm^2	0.0±0.0	1.1±1.1	6.7±3.3	8.9±2.6	10.0±2.9	16.7±8.5	73.3±5.8
	0.10 mg (a.i.)/cm^2	0.0±0.0	2.2±1.5	8.9±2.6	13.3±3.3	22.2±4.9	26.7±6.0	82.2±4.7
Sprayed surface	One surface is sprayed/ exposure onto sprayed surface							
Insecticide	Dose							
Thiamethoxam	0.025 mg (a.i.)/cm^2	0.0±0.0	0.0±0.0	2.2±2.2	26.7±3.3	52.2±4.7	67.8±4.0	84.4±2.9
	0.05 mg (a.i.)/cm^2	0.0±0.0	1.1±1.1	5.6±2.4	30.0±2.9	57.8±3.6	72.2±5.2	85.6±1.8
	0.10 mg (a.i.)/cm^2	0.0±0.0	2.2±2.2	5.6±2.9	36.7±3.7	58.9±3.5	73.3±2.9	90.0±2.9
Pirimiphos-methyl	0.025 mg (a.i.)/cm^2	0.0±0.0	0.0±0.0	0.0±0.0	1.1±1.1	16.7±4.4	52.2±3.2	100.0±0.0
	0.05 mg (a.i.)/cm^2	0.0±0.0	0.0±0.0	0.0±0.0	1.1±1.1	41.1±2.6	100.0±0.0	100.0±0.0
	0.10 mg (a.i.)/cm^2	0.0±0.0	0.0±0.0	0.0±0.0	2.2±2.2	42.2±3.6	100.0±0.0	100.0±0.0
alpha-cypermethrin	0.025 mg (a.i.)/cm^2	0.0±0.0	0.0±0.0	1.1±1.1	14.4±3.4	50.0±3.7	67.8±4.0	85.6±3.8

	0.05 mg (a.i.)/cm²	0.0±0.0	0.0±0.0	2.2±2.2	18.9±4.6	63.3±3.7	77.8±3.6	90.0±3.7
	0.10 mg (a.i.)/cm²	1.1±1.1	1.1±1.1	3.3±3.3	24.4±4.1	63.3±2.9	83.3±4.4	91.1±3.1
Deltamethrin	0.025 mg (a.i.)/cm²	0.0±0.0	0.0±0.0	5.6±2.9	8.9±3.9	15.6±5.0	15.6±5.0	70.0±7.1
	0.05 mg (a.i.)/cm²	1.1±1.1	1.1±1.1	6.7±2.4	14.4±2.9	16.7±4.1	24.4±5.3	72.2±6.0
	0.10 mg (a.i.)/cm²	0.0±0.0	0.0±0.0	12.2±2.2	23.3±4.7	24.4±4.4	38.9±7.5	81.1±6.6
Sprayed surface	Both surfaces are sprayed							
Insecticide	Dose							
Thiamethoxam	0.025 mg (a.i.)/cm²	0.0±0.0	0.0±0.0	0.0±0.0	2.2±1.5	2.2±1.5	6.7±1.7	8.9±1.1
	0.05 mg (a.i.)/cm²	0.0±0.0	0.0±0.0	0.0±0.0	3.3±1.7	5.6±2.4	11.1±3.1	25.6±2.4
	0.10 mg (a.i.)/cm²	0.0±0.0	0.0±0.0	0.0±0.0	4.4±4.8	15.6±4.1	34.4±4.1	48.9±3.5
Pirimiphos-methyl	0.025 mg (a.i.)/cm²	0.0±0.0	0.0±0.0	0.0±0.0	4.4±2.4	36.7±4.4	56.7±4.4	67.8±6.2
	0.05 mg (a.i.)/cm²	0.0±0.0	0.0±0.0	0.0±0.0	5.6±1.8	42.2±6.4	60.0±5.0	71.1±5.6
	0.10 mg (a.i.)/cm²	0.0±0.0	0.0±0.0	0.0±0.0	5.6±1.8	53.3±4.7	81.1±5.1	96.7±4.4
alpha-cypermethrin	0.025 mg (a.i.)/cm²	0.0±0.0	0.0±0.0	0.0±0.0	2.2±1.5	12.2±3.6	32.2±6.6	61.1±5.9
	0.05 mg (a.i.)/cm²	0.0±0.0	0.0±0.0	0.0±0.0	18.9±2.0	46.7±3.7	78.9±4.2	92.2±2.8
	0.10 mg (a.i.)/cm²	0.0±0.0	0.0±0.0	0.0±0.0	25.6±4.4	58.9±2.0	91.1±3.9	96.7±2.4
Deltamethrin	0.025 mg (a.i.)/cm²	0.0±0.0	0.0±0.0	0.0±0.0	13.3±3.3	28.9±5.4	35.6±4.4	63.3±5.5
	0.05 mg (a.i.)/cm²	0.0±0.0	0.0±0.0	1.1±1.1	14.4±3.8	32.2±4.3	41.1±4.8	65.6±6.0
	0.10 mg (a.i.)/cm²	0.0±0.0	0.0±0.0	5.6±3.4	20.0±3.7	32.2±4.0	42.2±5.2	67.8±4.0
